# Protein and Solvent Reorganization Drives Radical Pair Stability in Avian Cryptochrome 4a

**DOI:** 10.1021/jacs.5c15726

**Published:** 2025-11-14

**Authors:** Jiate Luo, Jonathan Hungerland, Ilia A. Solov’yov, Joseph E. Subotnik, Sharon Hammes-Schiffer

**Affiliations:** † Department of Chemistry, 6740 Princeton University, Princeton, New Jersey 08544, United States; ‡ Institut für Physik, 11233 Carl von Ossietzky-Universität Oldenburg, Carl-von-Ossietzky Str. 9-11, Oldenburg D-26129, Germany; § Research Center for Neurosensory, Science, Carl von Ossietzky Universität Oldenburg, Oldenburg 26111, Germany; ∥ Center for Nanoscale Dynamics (CENAD), Institute of Physics, Carl von Ossietzky Universität Oldenburg 26129 Oldenburg, Germany

## Abstract

Within the photoreceptor cells of avian retinas, cryptochrome-4a (Cry4a) has been proposed as a magnetic compass sensor in migratory songbirds. Recent photochemistry studies have demonstrated that Cry4a from the night-migratory European robin (*Erithacus rubecula*) exhibits greater magnetic sensitivity compared to the nonmigratory bird species. In *Er*Cry4a, blue-light excitation of the flavin triggers stepwise electron transfer along a tryptophan tetrad to the flavin, forming well-separated radical pairs that have been proposed to play a role in magnetoreception. Herein, we employ first-principles electronic structure methods and hybrid quantum mechanical/molecular mechanical (QM/MM) simulations to investigate the stabilization, interconversion, and recombination of these well-separated radical pairs. Reorganization of the protein environment and the aqueous solvent substantially stabilizes the long-range charge-transfer states associated with the anionic flavin radical and the cationic radical localized on the third or fourth tryptophan, rendering these radical-pair states energetically comparable to those in the charge-neutral state. Free energy analysis combined with electronic coupling calculations provides supporting evidence for the previously proposed idea of a “composite” radical pair, involving both the third and fourth tryptophans, as the functional magnetoreceptor. To provide guidance in probing the potential role of the composite radical pair in magnetoreception, we identify key amino acid residues whose mutation may significantly alter the relative stabilization and chemical dynamics of these radical pairs and consequently affect magnetic field sensitivity of *Er*Cry4a.

## Graphical abstract

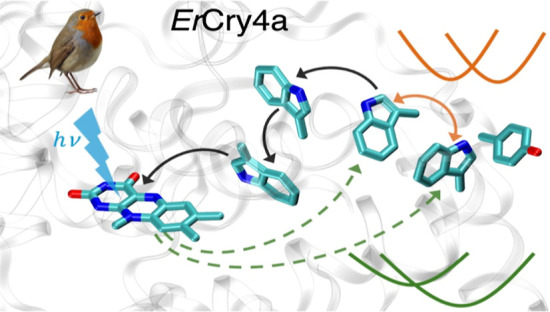

## Introduction

Cryptochromes (Cry), a class of flavoproteins, play a crucial role in various biological processes, including the regulation of circadian rhythms in animals and plants, as well as the control of plant growth and flowering.^1–3^ Moreover, cryptochromes have been proposed to be light-dependent magnetoreceptors that may endow night-migratory songbirds with the ability to detect the Earth’s magnetic field and utilize it for navigation over thousands of kilometers.^4–11^ Among the six Crys expressed in the avian retina,^12–23^ Cry1a and Cry4a are currently discussed as the primary contenders for functioning as magnetoreceptors.^24–26^ Because Cry1a exhibits a pronounced daily expression pattern^17^ and does not seem to strongly bind the essential flavin adenine dinucleotide (FAD) chromophore *in vitro*,^27–30^ Cry4a appears the most natural target to study. Further support for Cry4a as the target is its significantly higher expression during the migratory season^17^ and studies demonstrating a 97% probability of FAD binding upon recombinant protein expression and purification.^26^ Moreover, recent photochemistry experiments^26^ on purified Cry4a from the night-migratory European robin (*Erithacus rubecula*, *Er*) *in vitro* showed a spectroscopic signal that changes by application of ∼10 mT magnetic fields, an effect that is considerably larger than that observed with Cry4a from nonmigratory chicken (*Gallus gallus*, *Gg*) and pigeon (*Columba livia*, *Cl*). Thus, it is plausible to assume that *Er*Cry4a, with its enhanced magnetic sensing and signaling capabilities, is a suitable protein candidate for avian magnetoreception.^26^

The biophysics underlying the cryptochrome-based magnetoreceptor is intrinsically linked to the so-called radical pair mechanism,^5,10,31–37^ which proposes that weak magnetic field effects arise from different chemical reaction pathways available to different spin states within spin-correlated radical pairs.^35,38–46^ In *Er*Cry4a, the proposed magnetically sensitive radical pairs arise from sequential electron transfers along a chain of aromatic amino acid residues spanning from the FAD cofactor inside the protein to the protein surface.^19,26,47–49^ A brief description of the radical-pair formation is as follows and is illustrated in Figure 1:

I. Blue-light excitation of the fully oxidized FAD inside *Er*Cry4a initiates a rapid (subpicosecond) electron transfer from the nearby tryptophan W395 residue (denoted Trp_A_) to the photoexcited FAD*, creating the first radical pair, [FAD^•–^ Trp_A_H^•+^], denoted as RP_A_;^48^
II. A second electron transfer from W372 (denoted as Trp_B_) to the Trp_A_H^•+^ cation radical on a time scale of 30 ps outpaces the radical recombination of RP_A_, which occurs on a time scale of around 89 ps. This step leads to the creation of the second radical pair, [FAD^•–^ Trp_B_H^•+^], denoted as RP_B_;^48^
III. A further electron transfer from W318 (denoted as Trp_C_) to Trp_B_H^•+^ on a time scale of 140 ps is more than ten times faster than the geminate recombination of RP_B_, for which careful interpretation of 1.5 ns pump–probe experiments suggests a time scale of 1.6 ns.^48^ This step leads to the formation of the RP_C_, [FAD^•–^ Trp_C_H^•+^], inside *Er*Cry4a;
IV. The fast electron hopping between Trp_C_ and W369, denoted as Trp_D_, corresponding to Trp_C_H^•+^ + Trp_D_H *⇌* Trp_C_H + Trp_D_H^•+^, occurs with similar forward and reverse rate constants, leading to a dynamic equilibrium between RP_C_ and RP_D_.^26,47^ Although the 1.5 ns pump–probe experiments in ref 48 do not provide definitive recombination rates for the RP_C_ and RP_D_ states, μs pump–probe measurements^26^ suggest lifetimes of more than 1 μs for both states.

**Figure 1:**
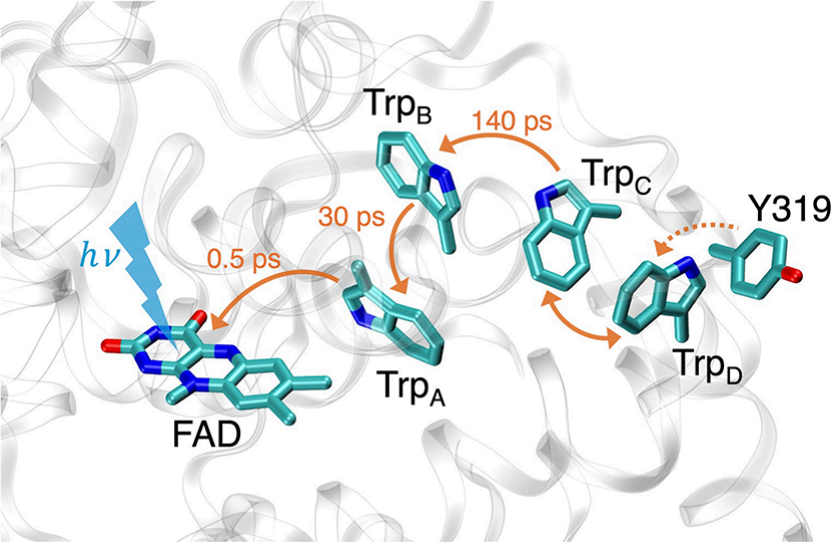
Depiction of the principal electron transfer pathway upon photoexcitation, involving the FAD chromophore, the tryptophan tetrad (solid orange arrows), and possibly Y319 (dashed orange arrow) in *Er*Cry4a. The time scales for the first three electron transfer steps are shown next to the solid orange arrows.

The rate of coherent singlet–triplet interconversion in radical pairs, driven by internal magnetic interactions such as the hyperfine couplings between the unpaired electron and the nuclear spins, can be modulated by the Zeeman interaction of the electron spin with external magnetic fields, thereby altering the likelihood of singlet radical recombination or the formation of alternative reaction products. A radical-pair lifetime exceeding the Larmor period of a precessing electron in the geomagnetic field (∼700 ns) is considered optimal for magnetic sensitivity.^5,50^ In particular, within a radical pair mechanism, efficient magnetic sensing requires spin-selective reactions to occur on a time scale competitive with the spin-independent reactions before spin relaxation disrupts coherent spin dynamics.^41,47,51–53^ For *Er*Cry4a, such a long-lived radical pair was proposed to involve RP_C_ and RP_D_.^26,47^ In RP_C_, the singlet radical recombination was proposed to compete with the deprotonation of the tryptophan cation radical on a microsecond time scale, allowing the geomagnetic field to effectively modulate radical-pair spin dynamics and influence product yield.^26,47^ On the other hand, RP_D_ has a larger separation between the radicals and therefore was suggested to have a prolonged lifetime, most likely on the order of milliseconds or longer, allowing FAD^•–^ to be stabilized via protonation to form the neutral semiquinone radical FADH^•^, thereby initiating a biochemical signaling cascade.^26^

Compelling evidence for steps (I), (II), and (III) above has been provided by ultrafast transient absorption spectroscopy carried out on *Er*Cry4a wild-type and site-specific mutants, whereby each tryptophan involved in the successive electron transfer was replaced with a redox-inactive phenylalanine.^48^ Direct evidence for the electron transfer between Trp_C_ and Trp_D_ could not be resolved experimentally.^48^ However, the enhanced level of the radical-pair concentration observed beyond several nanoseconds in wild-type *Er*Cry4a, compared to the Trp_D_ mutant where RP_D_ formation is precluded, strongly hints at a rapid equilibrium between RP_C_ and RP_D_, as suggested in (IV).

In addition to the ultrafast transient absorption spectroscopy results, the rapid equilibrium between RP_C_ and RP_D_ is also supported by other experimental observations. Electron paramagnetic resonance (EPR) experiments^26^ have shown that the radical pair formed in the wild-type *Er*Cry4a has a separation distance approximately 3 Å larger than in the Trp_D_ mutant, suggesting that the presence of Trp_D_ in the wild-type protein leads to one more sequential electron transfer and presumably a longer lifetime. These EPR experiments should be interpreted with caution, as they were conducted at a temperature as low as 80 K and cannot conclusively rule out a thermodynamically driven equilibrium between RP_C_ and RP_D_ under physiological conditions. Moreover, optical absorption studies have demonstrated that the radicals decay to the ground state with similar time constants in the cases of the wild-type protein and its Trp_D_ mutant,^26^ an observation that is consistent with nearly degenerate electron transfers between Trp_C_ and Trp_D_. Finally, although we will not pursue such a direction in this paper, beyond Trp_D_, we note that another sequential electron transfer involving a tyrosine residue (Y319) situated next to Trp_D_ is possible. It has been hypothesized that Y319 could possibly be reduced by the Trp_D_H^•+^ radical, and the resulting flavin-tyrosine radical pair is expected to recombine even slower than RP_D_.^47^ However, a long-lived tyrosine radical, reported in chicken Cry4a^54^ or implied in pigeon Cry4a,^55^ has not yet been observed in *Er*Cry4a for the photochemistry experiments conducted on the time scale of microseconds.^26^ Nevertheless, a lack of direct evidence does not entirely rule out the presence of a tyrosine radical, as the absorption band of TyrO^•^ is narrow and significantly obscured by the bands of the dark and radical states of the FAD and tryptophan.^56^

The goal of the present study is to focus on the well-separated RP_C_ and RP_D_, which are thought to be at the heart of the proposed avian magnetoreception mechanism.^26,47^ Our objective is to elucidate how RP_C_ and RP_D_ become stabilized, interconvert, and recombine, as fast equilibration between these states is essential for a radical pair magnetic field effect. Extending beyond the previous semiclassical analysis,^26,47^ we perform first-principles quantum chemical calculations of the electronic states for both dark state and radical-pair configurations in *Er*Cry4a. We evaluate the stabilization of charge separation within the long-range electron transfer pathway of the photocycle and examine the importance of associated conformational changes within and outside the active site of *Er*Cry4a. By analysis of the free energy profiles associated with the radical-pair reactions essential for magnetic sensitivity, we provide further validation for the hypothesized involvement of both RP_C_ and RP_D_ in magnetoreception through their rapid interconversion. Finally, a detailed electrostatic analysis is carried out to quantify the contributions from conformational changes in *Er*Cry4a and the surrounding solvent, identifying key amino acid residues that are essential for the stabilization and interconversion between the long-lived radical pairs. Our investigation lays the foundation for future mutation experiments aimed at testing the involvement and significance of the well-separated radical pairs in the proposed cryptochrome-based magnetoreception.

## Methods

Three 200 ns molecular dynamics (MD) trajectories corresponding to the dark-state (DS), the RP_C_, and the RP_D_ of *Er*Cry4a were obtained from an earlier study^26^ and used to sample conformations corresponding to these three different charge states. First-principles calculations were performed to compute the excitation energies for conformations selected along each MD trajectory to incorporate the effects of the conformational sampling. For these calculations, we employed a hybrid quantum mechanical/molecular mechanical (QM/MM) approach combined with either density functional theory (DFT) or linear-response time-dependent density functional theory (TDDFT) for the QM region, which was chosen to include the FAD and relevant protein residues. In addition, the diabatic energies and electronic couplings associated with the relevant electron transfer reactions were computed with constrained density functional theory with configuration interaction (CDFT-CI). More details about these methods are given in the remainder of this section.

### Molecular dynamics

The atomistic protein structure was obtained by homology modeling the sequence of *Er*Cry4a^17^ based on the pigeon Cry4a crystal structure^55^ using the Swiss-model.^57^ Due to the lack of terminal residues in the pigeon crystal structure, the *Er*Cry4a model only covers residues 8-495; most notably, this means the C-terminal tail has not been included. MD simulations were performed using NAMD2 ^58^ with the CHARMM36m force field for the protein^59–61^ with adapted parameters for the radical species of FAD^9^ and Trp.^34^ The appropriate parameters were chosen to represent the DS, RP_C_, and RP_D_ configurations. The protein was solvated in a water box of 92.08 × 104.86 × 100.31 Å, where the H_2_O molecules were modeled by using the TIP3P force field. 50 mM NaCl was added to reproduce the typical salt concentration in the experiments. Independent simulations were spawned for the DS, RP_C_, and RP_D_ configurations of the protein. A cutoff distance of 12 Å was used for explicit nonbonded interactions, and van der Waals forces were gradually switched off starting at 10 Å. Long-range electrostatics were computed every second step. The Langevin thermostat was applied only to non-hydrogen atoms with a damping coefficient of 5 ps^–1^. The barostat was set to decay within 50 fs and had an oscillation period of 200 fs. The equilibration started with energy minimization and 1 ns of MD simulation using 1 fs time step performed with the protein harmonically restrained while a Langevin-piston barostat kept the pressure at 1 atm and the Langevin dynamics modeled a temperature of 310 K to mimic the physiological temperature of avian cells. In the second equilibration phase, the backbone atoms of the protein were harmonically restrained for 3 ns. The last equilibration phase was carried out for 10 ns, where all restraints were released. All the production simulations were carried out for 200 ns using a time step of 2 fs and did not employ the barostat. Bond lengths involving hydrogen were constrained using SETTLE for water molecules and SHAKE for all other hydrogens.

### Hybrid QM/MM Approach

A hybrid QM/MM approach was adopted to include the effects of the solvated protein on the electronic states of the DS and the radical pair configurations. The QM region included the luminflavin moiety of the FAD chromophore and the side chains of the W395, W372, W318, W369, and Y319 residues (see Figure 1), as all are important for the sequential electron transfers.^26,47,50,62,63^ The MM region included the solvent and the remainder of the protein, including the phosphate group and the adenine nucleotide of the FAD cofactor, and the backbones of the four tryptophans and the tyrosine. The QM region was treated with electrostatic embedding by using the partial point charges from the force field used for the original MD simulations. The QM/MM interface in AMBER with the default charge redistribution scheme was used to add the terminal hydrogen link atoms between the C_β_ and C_α_ atoms of the tryptophan and tyrosine residues, as well as between the C1′ and C2′ atoms of the FAD. These link atoms are necessary to generate covalent bonds at the QM-MM boundary.

### Time-Dependent Density Functional Theory

Linear response TDDFT was employed to compute the first 20 electronic excitation energies for each conformation selected from the MD trajectories. These calculations used the ωB97X-D functional,^64^ which is a range-separated hybrid functional including a dispersion correction, and the 6-31+G** basis set for the QM region. The Tamm–Dancoff approximation (TDA)^65^ was adopted to avoid triplet instabilities. For all conformations from the DS trajectory, the ground state has closed-shell character, and restricted DFT orbitals were used as the reference for the TDA-TDDFT calculations. For the RP_C_ and RP_D_ trajectories, the ground state was not necessarily of closed-shell character; for some configurations, the ground state was an open-shell charge-transfer (CT) state, as found by an unrestricted DFT calculation. In all cases, TDDFT calculations were built on top of the true, stable DFT minimum energy solution, which was not always a restricted solution.

It is important to note that standard TDA-TDDFT is based on a single reference configuration and therefore does not accurately describe systems with significant multireference ground-state character. Spin-flip (SF) TDA-TDDFT, starting from a high-spin (triplet) reference and including a single α → β spin flip, offers a more suitable approach when the ground state appears to be an open-shell singlet diradical.^66^ Therefore, conformations with an open-shell CT ground state were studied using both standard TDA-TDDFT and SF-TDA-TDDFT approaches; the results from the two methods were compared and found to be identical. Hence, all of the results discussed below correspond to the standard TDA-TDDFT methodology. In addition, the energies of the first excited charge-transfer state relative to the charge-neutral state for conformations selected from the RP_C_ and RP_D_ trajectories are similar when computed with TDA-TDDFT and ΔSCF (Section B in the Supporting Information).

### Constrained Density Functional Theory with Configuration Interaction

The QM/MM CDFT-CI method^67^ was used to construct the diabatic states and to compute the electronic couplings for the long-range electron transfer processes. Specifically, 800 conformations were extracted at 0.25 ns intervals from each of the MD trajectories equilibrated and propagated in the DS, RP_C_, and RP_D_ configurations. Equally spaced trajectory subsampling delivers a reasonable set of varying atomic configurations without introducing the need for a postprocessing reweighting procedure. CDFT enforces an explicit constraint to force the charge or spin to localize on specified fragments. Spin constraints were imposed to study the charge-neutral, CT_C_ (i.e., [FAD^•–^ Trp_C_H^•+^]), and CT_D_ (i.e., [FAD^•–^ Trp_D_H^•+^]) states. For the charge-neutral state, the net spin density was zero for every molecular fragment in the QM region. For the CT_C_ state, a constraint was applied to maintain a spin of +1/2 on FAD^•–^ and −1/2 on Trp_C_H^•+^. For the CT_D_ state, a constraint was applied to maintain a spin of +1/2 on FAD^•–^ and −1/2 on Trp_D_H^•+^. The ωB97X-D functional^64^ and 6-31+G** basis set were used for these calculations. Within the CDFT-CI framework, a 3 × 3 CI matrix was built for the three diabatic states in a nonorthogonal basis. Löwdin symmetric orthogonalization was used to transform the CI matrix to the orthogonal basis. The electronic couplings are the off-diagonal elements of the CI matrix in the orthogonal basis.

## Results and Discussion

### Impact of Conformational Changes on Excitation Energies

In *Er*Cry4a, photoexcitation of the flavin leads to conformational changes within the protein that induce subsequent sequential electron transfer reactions, which are accompanied by further conformational changes. The MD trajectories corresponding to the DS, RP_C_, and RP_D_ configurations provide insights into the conformational changes of the active site and protein and solvent environments that occur in response to the redistribution of charges associated with the well-separated radical pairs. In this section, we examine the energetic impact of these conformational changes, and in a later section we will perform an electrostatic analysis to identify the key conformational changes. To examine the impact of the conformational changes on the excitation energies, we performed single-point TDA-TDDFT/ωB97X-D/6-31+G** calculations on the active site in the gas phase (i.e., only the QM region) and with electrostatic embedding to incorporate the partial charges of the protein and the aqueous environment. These calculations were performed for conformations along the DS, RP_C_, and RP_D_ trajectories to probe protein environments that are equilibrated to the neutral state and to each of the two radical-pair states.

The natural transition orbitals (NTOs) obtained from the TDA-TDDFT calculations with electrostatic embedding elucidate the characteristics of the excited states. The NTOs of the first singlet excited state of representative conformations selected from the DS, RP_C_, and RP_D_ trajectories illustrate the significant differences in the characters of these first excited states. For each conformation plotted in Figure 2, the ground state is closed shell and charge neutral, and the first excited singlet state corresponds to the local excitation of the FAD cofactor for the DS trajectory or to the electronic state maintained during the MD trajectory for the RP_C_ and RP_D_ trajectories. Specifically, the first excited singlet state corresponds to the CT states associated with charge transfer from Trp_C_ or Trp_D_ to FAD for the RP_C_ and RP_D_ trajectories, respectively. The lowest 20 states obtained from the TDA-TDDFT calculations for the three conformations, either in the gas phase or with electrostatic embedding, are summarized in Table S1 of the Supporting Information. The energies of the lowest locally excited FAD states are all around 3.0 eV (see Table S1), in good agreement with the experimentally measured value of ∼2.8 eV for the flavin excitation. The local FAD excitation remains largely unaffected by the conformational changes in the active site of *Er*Cry4a or the rearrangement of the electrostatic environment, similar to other flavin-containing photoreceptors, such as the plant cryptochrome^9^ and BLUF.^68^

**Figure 2:**
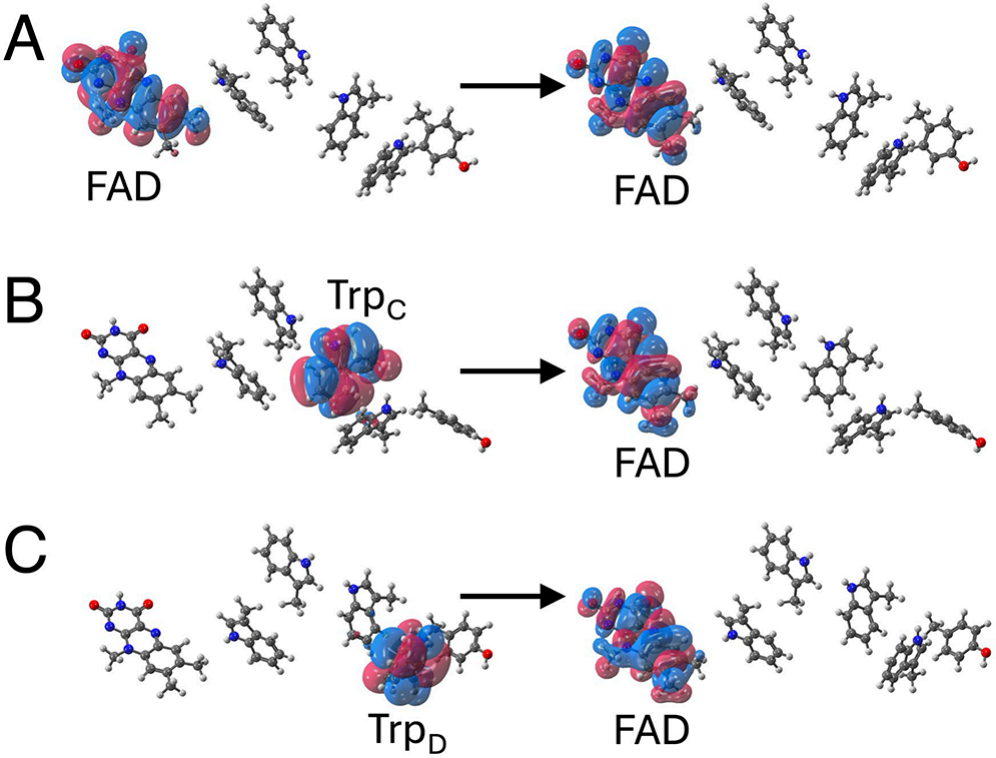
Dominant natural transition orbitals (NTOs) illustrating transitions from an occupied to a virtual orbital characterizing the first excited state, as obtained from TDA-TDDFT with electrostatic embedding for three representative conformations. (A) Representative conformation obtained from the DS trajectory, where the first excited state corresponds to a local excitation of the FAD. (B) Representative conformation obtained from the RP_C_ trajectory, where the first excited state corresponds to charge transfer from Trp_C_ to the FAD. (C) Representative conformation obtained from the RP_D_ trajectory, where the first excited state corresponds to charge transfer from Trp_D_ to FAD.

In contrast, the energies of the CT states are sensitive to the electrostatic environment (Figure 2). To further analyze this sensitivity, we sampled multiple conformations by selecting 40 conformations at 5 ns intervals from each of the DS, RP_C_, and RP_D_ trajectories. For each conformation, we computed the excitation energies with TDA-TDDFT for the QM region in the gas phase or including either only the protein or both the protein and solvent environment with electrostatic embedding. Overall, the inclusion of both the protein and solvent environments induces a substantial shift in energy levels. For all 40 conformations sampled from the RP_C_ trajectory, the CT_C_ state appears as either the first excited (S1) state (21 out of 40 conformations), as shown in Figure 2B, or as the ground state (19 out of 40 conformations). Similarly, for all 40 conformations sampled from the RP_D_ trajectory, the CT_D_ state appears as either the S1 state (15 out of 40 conformations), as shown in Figure 2C, or the ground state (25 out of 40 conformations). For conformations with a charge-transfer ground state, the closed-shell charge-neutral state shifts to an excited state. We focus on the energies of the lowest CT_B_, CT_C_, and CT_D_ states relative to the charge-neutral state, defined as Δ*E*_X_ = *E*_X_ – *E*_neutral_ for X ∈ {CT_B_, CT_C_, and CT_D_}. The charge-neutral or charge-transfer character of the ground and excited states was identified by the natural charges obtained from a natural bond orbital analysis. Figure 3 shows the average relative energies, ⟨Δ*E*_X_⟩, and the corresponding standard deviations, σ­(Δ*E*_X_), which reflect the spread of Δ*E*_X_.

**Figure 3:**
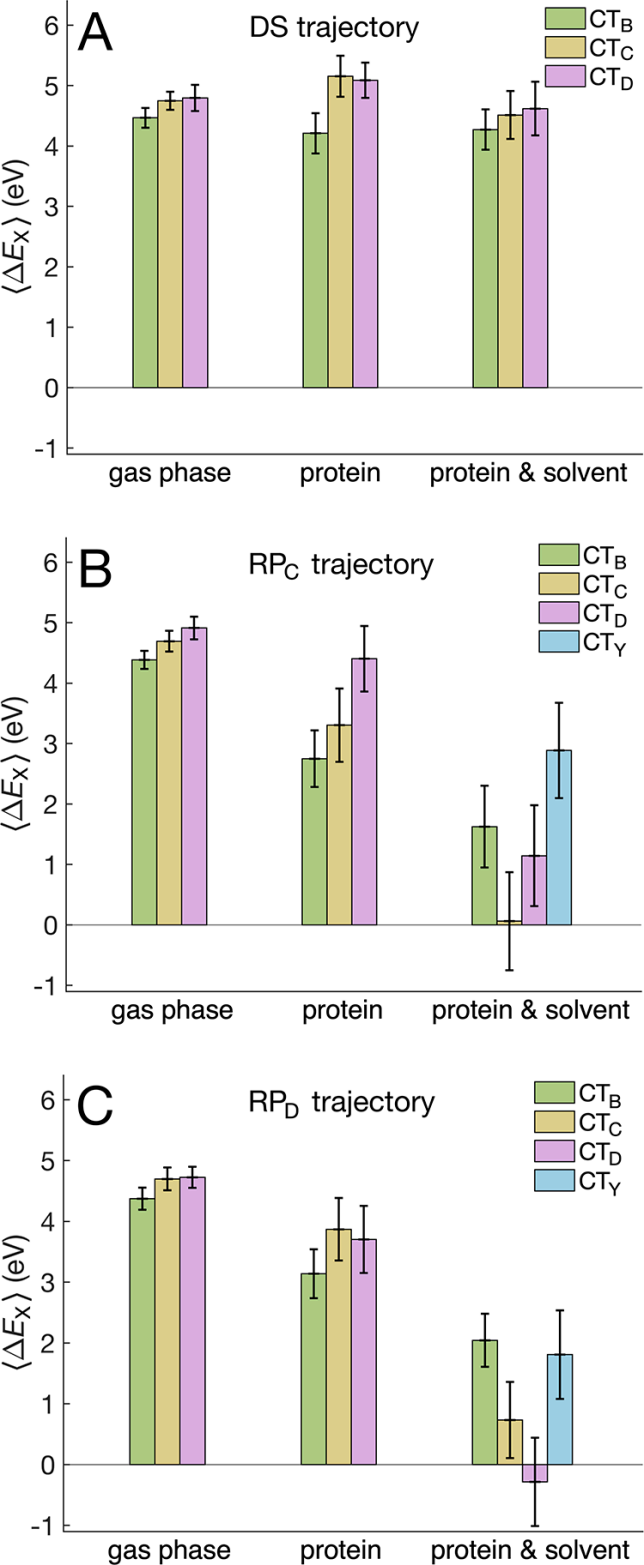
Average relative energies of the charge-transfer states compared to the charge-neutral state, as obtained from TDA-TDDFT calculations for conformations sampled from trajectories representing (A) the DS, (B) the RP_C_ state, and (C) the RP_D_ state. The “gas phase” panels represent calculations of the QM region in vacuum; the “protein” panels represent QM/MM calculations including the protein environment with electrostatic embedding; the “protein and solvent” panels represent QM/MM calculations including the protein and solvent environment with electrostatic embedding. The error bars indicate the standard deviations, illustrating the spread of the relative energies. The relative energies of the CT_A_ state compared to the charge-neutral state are outside the scope of this work and are therefore omitted from the figure for clarity.

Gas-phase calculations of the QM region produce similar $\left\langle \Delta E_{{CT}_{B}}\right\rangle$ , $\left\langle \Delta E_{{CT}_{C}}\right\rangle$ , and $\left\langle \Delta E_{{CT}_{D}}\right\rangle$ values for conformations sampled from the DS, RP_C_, and RP_D_ trajectories (compare the “gas phase” panels in Figure 3A–C). These CT excitation energies are all significantly higher than the local flavin excitation energy of 3.0 eV. In addition, the corresponding σ­(Δ*E*_X_) values for the three trajectories are similar and appear to be quite small, indicating minimal variation of the excitation energies within each trajectory. Although variations in the distances and orientations among the FAD, the tryptophan tetrad, and the tyrosine during the sequential electron transfer process have been observed in ref 26, conformational changes within the active site alone are insufficient to energetically stabilize the long-range CT states. Thus, the effects of the environment are expected to be critical in facilitating the efficient formation and stabilization of the long-lived radical pair, as also discussed earlier.^69–72^

To that end, the impact of the protein environment is clearly shown in the middle (“protein”) panels in Figure 3. For the DS trajectory (Figure 3A), the inclusion of the electrostatic protein environment has a relatively small impact on the excitation energies, although the energy distribution becomes broader. More significant environmental effects become visible for the conformations sampled from the RP_C_ and RP_D_ trajectories. Including only the electrostatic protein environment leads to a pronounced reduction in the excitation energies of the CT states. Perhaps surprisingly, the CT_B_ state exhibits the greatest decrease in excitation energy and appears to be the lowest in energy among the considered CT states for both radical-pair configurations. Without considering the aqueous solvent, clearly the RP_C_ or RP_D_ configurations could only be transient intermediates in the photocycle of *Er*Cry4a and would be unlikely to be long-lived, magnetically sensitive radical pairs.

Finally, we examine the importance of the solvent environment, which has been shown to be critical for the formation of persistent well-separated radical pairs in earlier studies on plant cryptochromes.^10,34,73^ A depiction of the solvent exposure for the key active-site entities is provided in Figure S2 of Supporting Information. Inclusion of the solvent environment, together with the protein environment, has a relatively small impact on the excitation energies for the DS trajectory, although the energy distribution becomes even broader. In both radical-pair trajectories, the CT_C_ and CT_D_ states are stabilized by solvent to a much greater extent than is the CT_B_ state, presumably due to greater solvent exposure of Trp_C_ and Trp_D_. This solvent stabilization exceeds 3 eV and alters the ordering of the electronic states. For the RP_C(D)_ trajectory, the energy of the CT_C(D)_ state becomes comparable to the energy of the charge-neutral state, suggesting facile radical recombination of both RP_C_ and RP_D_, as discussed in detail in the following section. In combination with protein reorganization, solvent reorganization can preferentially stabilize the hole on Trp_C_ or Trp_D_, supporting a plausible exchange between RP_C_ and RP_D_.

Interestingly, the CT_Y_ state, corresponding to charge transfer from the tyrosine Y319 residue to the FAD, becomes much lower in energy once the combined impact of both the protein and solvent environments is included for the radical-pair trajectories (blue bars in Figure 3B,C). The average relative energy is lower in the RP_D_ trajectory (1.81 eV) than that in the RP_C_ trajectory (2.89 eV). Although reduction of Trp_C_H^•+^ by Y319 is unlikely given the large distance, the possibility of an electron transfer from Y319 to Trp_D_H^•+^ cannot be completely excluded,^47^ particularly considering that a coupled proton transfer from the surface-exposed Y319 residue to surrounding water could lower the associated free energy barrier.

### Free Energy Profiles and Kinetics for Radical-Pair Reactions

To investigate the kinetics of electron transfer between Trp_C_ and Trp_D_, as well as the recombination of the RP_C_ and RP_D_ radical pairs, we analyze the corresponding free energy profiles. These profiles were obtained by sampling the energy gaps between the reactant and product diabatic states for each electron transfer reaction. Specifically, QM/MM CDFT-CI calculations were performed on 800 conformations obtained at 0.25 ns intervals for each of the DS, RP_C_, and RP_D_ trajectories to obtain the energies of the diabatic charge neutral, CT_C_, and CT_D_ states. The sampled energy gaps between any two of these diabatic states approximately represent a Gaussian distribution, yielding probability density functions of the form

1 $$P\left(\Delta E\right)=\frac{1}{\sqrt{2\pi \sigma^{2}}}\exp\left(-\frac{{\left(\Delta E-\left\langle \Delta E\right\rangle \right)}^{2}}{2\sigma^{2}}\right)$$

where ⟨Δ*E*⟩ and σ are the average and standard deviation, respectively, of the energy gap sampled. The parabolic free energy surfaces along the energy gap coordinate Δ*E* are

2 $$G\left(\Delta E\right)=-k_{B}T\;\;\ln\left(P\left(\Delta E\right)\right)+G^{{}^{\circ}}$$

Figure 4 shows the Gaussian fits of the energy gap distributions, as well as the corresponding free energy profiles for each of the three electron transfer reactions considered. We constructed the parabolas using a value for σ^2^ that is the average of the values obtained from the Gaussian fits of the two distributions. *G*° was then determined by ensuring that the two free energy profiles cross at Δ*E* = 0, and the reorganization energy λ_r_ was determined from the resulting free energy profiles. Specifically, λ_r_ = *G*_X_(⟨Δ*E*⟩_Y_) – *G*_X_(⟨Δ*E*⟩_X_), where X and Y specify the state. Due to incomplete sampling and nonergodic dynamics in protein environments, care must be taken when evaluating and interpreting free energy surfaces. In particular, as discussed clearly in a previous study,^74^ the reorganization energy can be defined in several different ways. For more details and a brief review of how to compute the relevant reorganization energies in nonergodic protein environments, see the Supporting Information.

**Figure 4:**
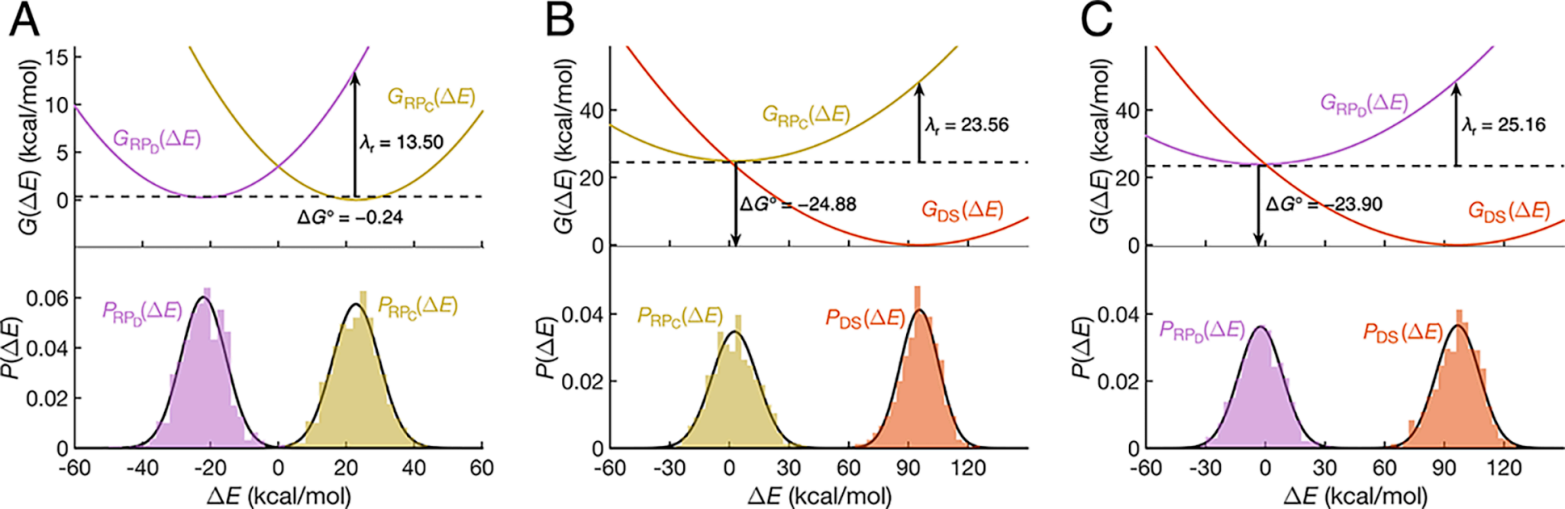
Distributions of energy gaps between relevant diabatic states and the corresponding free energy profiles for (A) electron transfer between Trp_C_ and Trp_D_; (B) radical recombination of RP_C_; and (C) radical recombination of RP_D_. The values of the effective reaction free energy Δ*G*° and reorganization energy λ_r_ are indicated in the figures. For (A), the energy gap Δ*E* is the energy difference between the CT_D_ and CT_C_ diabatic states, and the conformations are sampled over the RP_C_ (beige) and RP_D_ (purple) trajectories. For (B), the energy gap is the energy difference between the CT_C_ and charge-neutral states, and the conformations are sampled over the RP_C_ (beige) and DS (orange) trajectories. For (C), the energy gap is the energy difference between the CT_D_ and charge-neutral states, and the conformations are sampled over the RP_D_ (purple) and DS (orange) trajectories.

Beyond the free energy profile, the calculation of electron transfer rate constants requires the diabatic electronic coupling for electron transfer between Trp_C_ and Trp_D_ as well as between each of these tryptophans and FAD^•–^. Using CDFT-CI, we calculated the electronic couplings *T*_*ij*_, where *i* and *j* denote the corresponding diabatic states (see Figure 5). Within Marcus theory, electron transfer occurs at the crossing point of the two Marcus parabolas, which is where the energies of the two diabatic states are degenerate. The Marcus theory rate constant for nonadiabatic electron transfer is based on Fermi’s golden rule, which requires the energies of the two diabatic states to be degenerate for electron transfer to occur with a tunneling probability proportional to the square of the electronic coupling, $|T_{ij}{|}^{2}$ . Thus, the magnitude of $|T_{ij}{|}^{2}$ at the crossing point (i.e., where Δ*E* = 0), is most relevant to the electron transfer rate constant. In practice, we consider the electronic coupling for nuclear conformations corresponding to energy gaps in the relatively small range of −7 to 7 kcal/mol about Δ*E* = 0.

**Figure 5:**
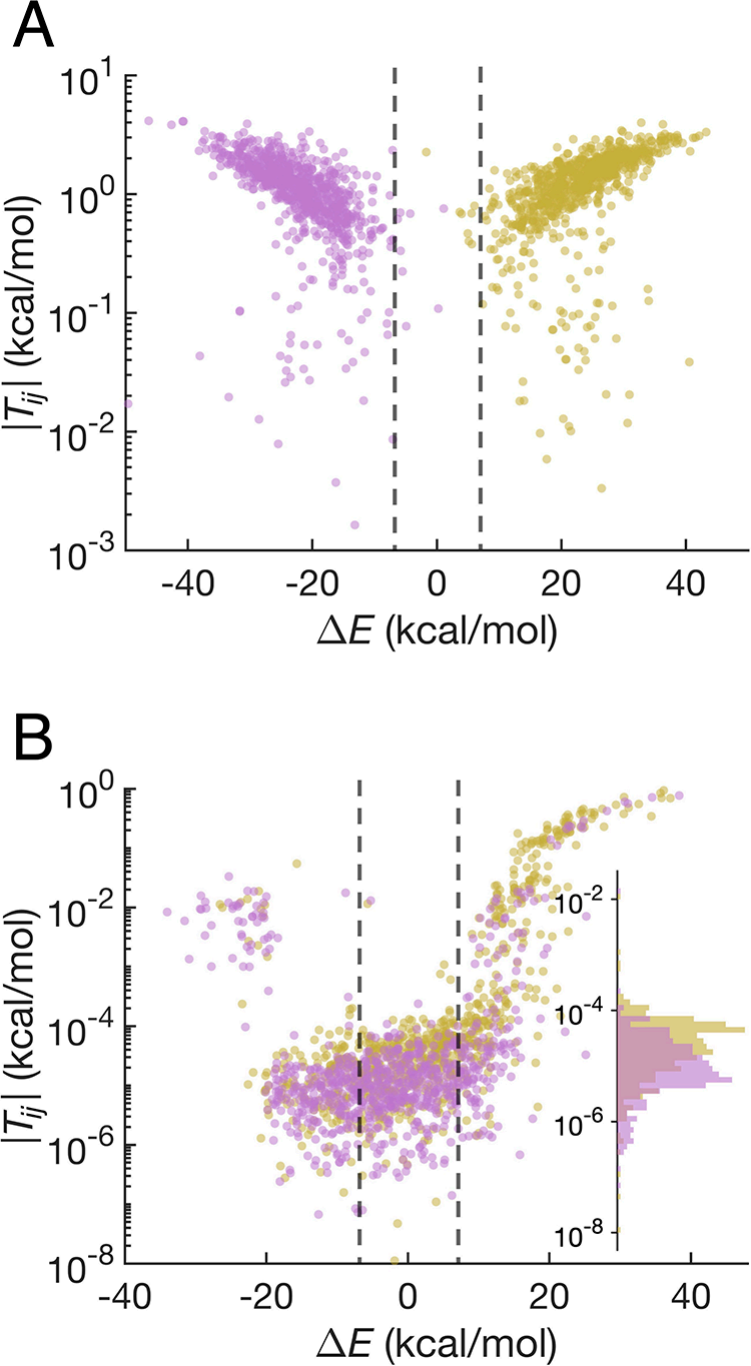
Electronic couplings between relevant diabatic states with respect to energy gaps for conformations sampled from MD trajectories. (A) Magnitudes of electronic couplings between the CT_C_ and CT_D_ states in RP_C_ (beige dots) and RP_D_ (purple dots) trajectories. (B) Magnitudes of electronic couplings between the CT_C_ and charge-neutral states in the RP_C_ trajectory (beige dots) and between the CT_D_ and charge-neutral states in the RP_D_ trajectory (purple dots). The inset compares the distributions of |*T*_*ij*_| for energy gaps in the range of −7 to 7 kcal/mol in RP_C_ (beige histogram) and RP_D_ (purple histogram) trajectories.

In order to verify the model for magnetoreception described in the Introduction, an estimate of the time scales for the electron transfer reactions involving the Trp_C_ and Trp_D_ radicals is essential. We have isolated 16 conformations within the dashed lines of Figure 5A, where the energy gap lies in the range of −7 to 7 kcal/mol. Given the reasonably large electronic couplings spanning 0.08 to 2.3 kcal/mol and a low barrier on the order of 3.3 kcal/mol (see Figure 4A), equilibration between these two diabatic states is expected to be quite rapid. The broad range of electronic couplings spans the electronically adiabatic and nonadiabatic regimes of electron transfer, given the thermal energy of 0.62 kcal/mol at *T* = 310 K. If the reaction were adiabatic, classical transition state theory would yield a rate constant greater than 1 × 10^10^ s^–1^. Alternatively, if the reaction were nonadiabatic, the rate constant could be estimated using the nonadiabatic electron transfer rate constant expression^75^

3 $$k=\frac{2\pi }{\hbar }{\left|T_{ij}\right|}^{2}\frac{1}{\sqrt{4\pi \lambda_{r}k_{B}T}}\exp\left(-\frac{{\left(\lambda_{r}+\Delta G^{{}^{\circ}}\right)}^{2}}{4\lambda_{r}k_{B}T}\right)$$

Analyzing the data to obtain a reaction free energy of Δ*G*° = −0.24 kcal/mol and a reorganization energy of 13.5 kcal/mol (Figure 4A), the nonadiabatic rate constant would be in the range of 10^9^–10^10^ s^–1^. In either case, the nearly degenerate electron transfer between Trp_C_ and Trp_D_ is expected to occur much faster than the period of the coherent singlet–triplet interconversion of flavin−tryptophan radical pairs in *Er*Cry4, which is typically greater than 10 ns.^5,11,52,76^ Note that a very recent computational study reported RP_D_ to be more thermodynamically favorable than RP_C_ by 3.23 kcal/mol, most likely due to differences in the starting protein structures and to a lesser degree also due to differences in computational methods.^77^ Interestingly, despite the difference in reaction free energy, this study^77^ reported a nonadiabatic rate constant of 7 × 10^9^ s^–1^, which agrees with the range reported here.

Finally, we consider the radical recombination reaction. The free energy parabolas cross near the minimum of *G*_RP_C(D)__ (Figure 4B,C), resulting in nearly barrierless reactions. The efficiency of radical recombination is thus primarily determined by the electronic couplings between the CT and the charge-neutral states. As shown in Figure 5B, for energy gaps in the range of −7 to 7 kcal/mol, the electronic couplings for both RP_C_ (beige dots) and RP_D_ (purple dots) recombinations are quite small, in the range of 10^–6^ to 10^–4^ kcal/mol. Given such small electronic couplings, the reaction can be assumed to be nonadiabatic, with corresponding lifetimes from 3 μs to 30 ms estimated by eq 3. It is important to note that the median electronic coupling associated with RP_C_ recombination is larger than that for RP_D_ recombination, as evidenced by the statistical separation between their distributions (see the insets in Figure 5B). Specifically, the median |*T*_*ij*_| for RP_C_ is 2.4 × 10^–5^ kcal/mol, which is approximately three times greater than the median of 9.0 × 10^–6^ kcal/mol for RP_D_ recombination. Hence, an order-of-magnitude longer lifetime can reasonably be expected for RP_D_ compared to RP_C_.

Overall, the rapid interconversion between RP_C_ and RP_D_ supports the recently proposed idea of a “composite” radical pair, which may offer better magnetic sensitivity than a single radical pair species.^26,47^ For a radical pair to function as an effective magnetoreceptor, its optimal lifetime must satisfy two conditions: it should be no longer than the spin relaxation time and no shorter than the Larmor period (∼700 ns) of the geomagnetic field, thereby ensuring that coherent spin dynamics can be sufficiently modulated by Earth’s magnetic field. The spin relaxation time in cryptochromes was estimated to be several μs in previous studies.^51,52,78,79^ Considering the higher end of the computed electronic couplings, this range of time scales is more likely to be met by RP_C_ than by RP_D_ recombination.

Alternatively, in order to satisfy the time scale requirements above, there is also the possibility that rather than the direct recombination FAD^•–^ Trp_C_H^•+^ → FAD Trp_C_H, instead RP_C_ recombines in a two-step process through RP_B_

$${\mathrm{FAD}}^{•-}\;{\mathrm{Trp}}_{B}H\;{\mathrm{Trp}}_{C}H^{•+}\overset{k_{\mathrm{CB}}}{\underset{k_{\mathrm{BC}}}{⇌}}\;{\mathrm{FAD}}^{•-}\;{\mathrm{Trp}}_{B}H^{•+}\;{\mathrm{Trp}}_{C}H\overset{k_{B}^{r}}{\to }\mathrm{FAD}\;{\mathrm{Trp}}_{B}H\;{\mathrm{Trp}}_{C}H$$

where *k*_CB_ and *k*_BC_ are the rate constants for RP_C_ → RP_B_ and RP_B_ → RP_C_, respectively, and *k*_B_^r^ is the rate constant for RP_B_ recombination. As noted in the Introduction, the ultrafast transient absorption spectroscopy indicates that *k*_B_^r^ ≈ 6.3 × 10^8^ s^–1^ and *k*_BC_ ≈ 7.1 × 10^9^ s^–1^. Although *k*_CB_/*k*_BC_ could be estimated from an equilibrium simulation, here we adopt the previously suggested *k*_CB_ based on the time dependence of magnetic field effects in *Er*Cry4a, which is in the range of 3–6 × 10^7^ s^–1^,^78^ and the time scale for this indirect pathway is thus estimated to be around several hundred ns.

### Electrostatic Analysis Probing Key Conformational Changes Stabilizing Radical Pairs

We employed electrostatic analysis to gain further insights into the specific conformational changes of both the protein and solvent that stabilize the radical pairs and facilitate the electron transfer reactions. For this purpose, we computed the electrostatic potential difference between the centers of the flavin, Trp_C_, and Trp_D_ for the DS, RP_C_, and RP_D_ trajectories. Here, we averaged over the same 800 conformations per MD trajectory that were used to generate the free energy distributions. The center points are defined as the geometric center of the isoalloxazine ring (midpoint between N5 and N10) of the flavin, denoted as **r**_F_, the centers of the indole rings of Trp_C_, denoted as **r**_C_, and Trp_D_, denoted as **r**_D_. The electrostatic potential *V* is defined as

4 $$V_{\left(r_{m}\right)}=\frac{1}{4\pi \varepsilon_{0}}\sum_{n}\frac{q_{n}}{\left|r_{m}-r_{n}\right|}$$

where ε_0_ is the vacuum permittivity, *q*_*n*_ is the partial charge of the *n*-th atom located at **r**_*n*_ within the MM subsystem, and **r**_*m*_ is the reference point, which could be **r**_F_, **r**_C_, or **r**_D_.

We first examine the electrostatic potential difference Δ*V* between Trp_C_ and Trp_D_

5 $$\Delta V_{\left(C-D\right)}=V_{\left(r_{C}\right)}-V_{\left(r_{D}\right)}$$

to investigate how the environment reorganizes for the hole transfer between Trp_C_ and Trp_D_. A negative Δ*V*_(C–D)_ value indicates that the hole is preferentially localized on Trp_C_ rather than Trp_D_, and vice versa for a positive value. As shown in Table 1, for the RP_C_ trajectory, the protein environment contributes a Δ*V*_(C–D)_ of −0.92 V, predominantly favoring localization of the hole on Trp_C_, whereas the solvent does not exhibit a strong electrostatic effect favoring either Trp_C_ or Trp_D_. As the hole transfers to Trp_D_, corresponding to the RP_D_ trajectory, both the protein and solvent reorganize to favor the hole on Trp_D_, owing to its greater solvent exposure near the protein surface. The total shift in Δ*V*_(C–D)_ for the hole transferring from Trp_C_ to Trp_D_ is given by

6 $$\Delta \Delta V_{\left(C-D\right)}=\Delta V_{\left(C-D\right)}^{{RP}_{D}}-\Delta V_{\left(C-D\right)}^{{RP}_{C}}$$

where the superscript indicates the trajectory that is sampled to obtain the electrostatic potential difference. The combined reorganization of the protein and solvent leads to ΔΔ*V*_(C–D)_ ≈ 2 V as the hole transfers from Trp_C_ to Trp_D_ (see Table 1). Figure 6A provides a detailed analysis of the protein reorganization contributing to ΔΔ*V*_(C–D)_. Seven key residues are identified, namely, I316, C317, K320, R324, T364, H477, and N478, located ∼3–16 Å away from Trp_C_ or Trp_D_. These residues collectively account for 77% of the protein reorganization contributing to ΔΔ*V*_(C–D)_. The spatial arrangement of these residues is depicted in Figure 7A. K320 made the most significant contribution. The ε-group NH_3_^+^ of K320 that carries a positive charge shows a strong inclination to reposition depending on the radical location. When Trp_C_ has a cation radical character, the corresponding N atom of K320 remains at a distance of approximately 15.5 Å from the center of Trp_C_. As the hole transfers to Trp_D_, this distance reduces to approximately 9.5 Å and K320 reorients.

**Table 1:** Electrostatic Potential Differences (in Volts) between Trp_C_ and Trp_D_, FAD and Trp_C_, and FAD and Trp_D_ Averaged over Conformations from the Trajectories Identified as DS, RP_C_, or RP_D_

	protein	solvent	total
$\Delta V_{\left(C-D\right)}^{{RP}_{C}}$	–0.92	0.09	–0.83
$\Delta V_{\left(C-D\right)}^{{RP}_{D}}$	0.36	0.88	1.24
ΔΔ*V*_(C–D)_	1.28	0.79	2.07
Δ*V*_(F–C)_^DS^	0.33	0.79	1.12
$\Delta V_{\left(F-C\right)}^{{RP}_{C}}$	2.70	2.83	5.53
ΔΔ*V*_(F–C)_	2.37	2.04	4.41
Δ*V*_(F–D)_^DS^	0.36	0.81	1.17
$\Delta V_{\left(F-D\right)}^{{RP}_{D}}$	2.18	3.61	5.79
ΔΔ*V*_(F–D)_	1.82	2.80	4.62

**Figure 6:**
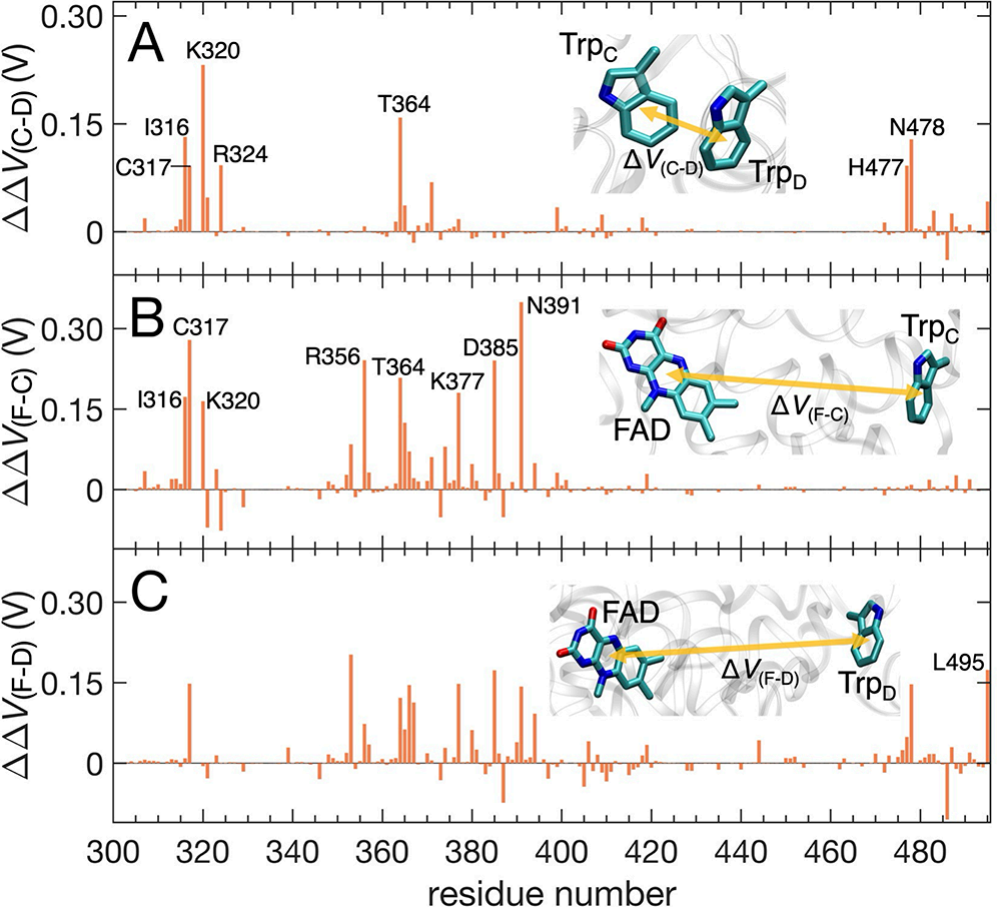
Contribution from each residue of *Er*Cry4a to the change in the electrostatic potential difference for (A) the hole transferring from Trp_C_ to Trp_D_ (ΔΔ*V*_(C–D)_ in eq 6); (B) the stabilization of RP_C_ relative to the DS (ΔΔ*V*_(F–C)_ in eq 8a); and (C) the stabilization of RP_D_ relative to the DS (ΔΔ*V*_(F–D)_ in eq 8b). Results are shown only for residues 300–495, as residues preceding 300 contribute minimally to ΔΔ*V*. The residue-specific contributions for the remaining residues are provided in the Supporting Information.

**Figure 7:**
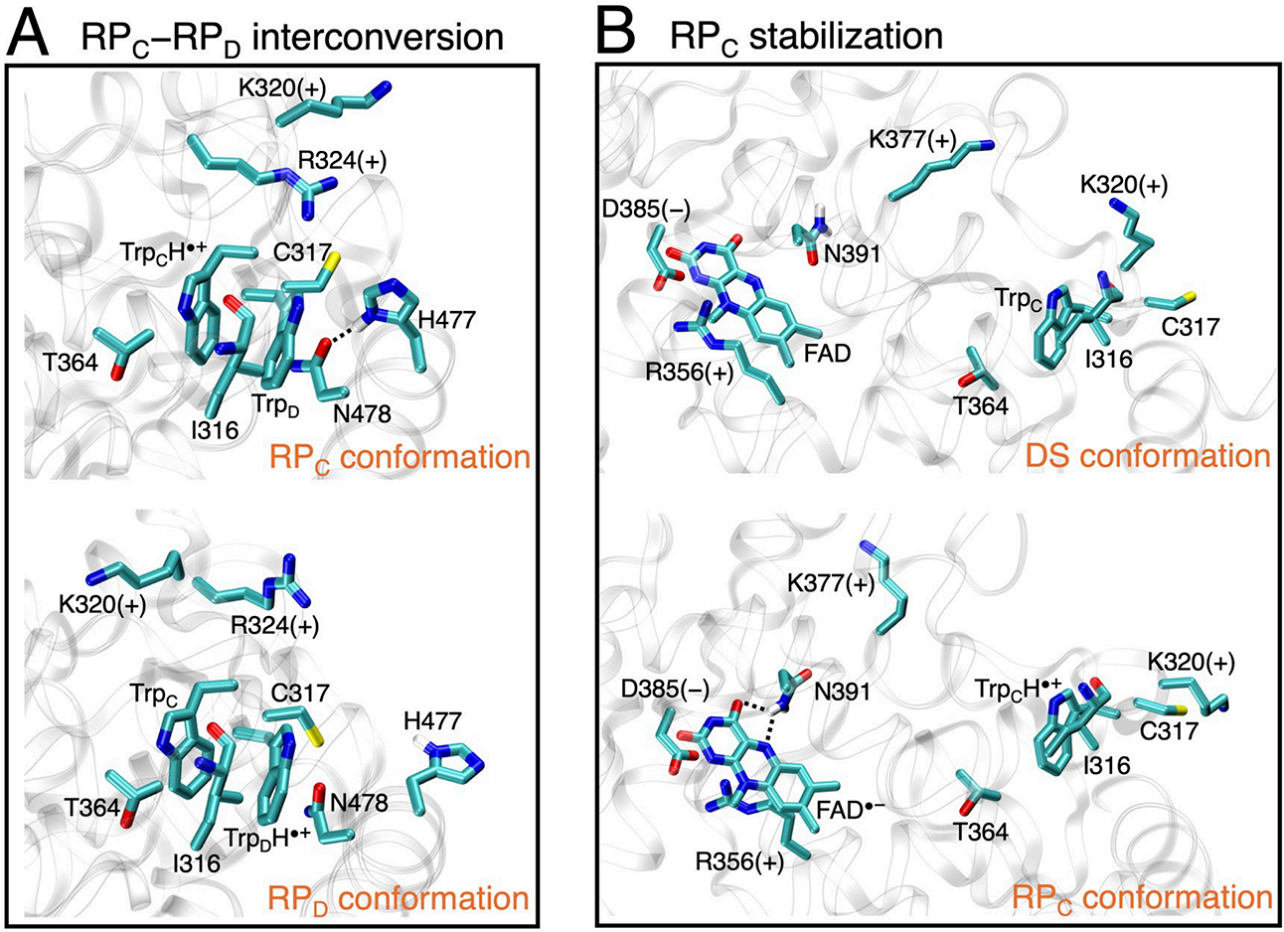
Depiction of key residues with significant impact on tuning the electrostatic potentials for (A) electron transfer between Trp_C_ and Trp_D_ and (B) the stabilization of RP_C_. All hydrogens, except for HD21 and HD22 in N391 and HD1 in H477, have been removed for clarity. Aside from I316, which modulates the electrostatic potential by backbone movement, the key residues are either charged or polar. In part (A), the charged residues, such as K320 and R324, shift their positions depending on whether the cation radical is on Trp_C_ or Trp_D_. The polar residues, such as C317, T364, N391, N478, and H477, modulate the electrostatic potential primarily through reorientation of their side-chain dipoles. Notably, H477 tends to be positioned closer to Trp_D_ in RP_C_ than in RP_D_, making it more likely to form hydrogen bonds with N478. In part (B), positively charged residues, such as R356 and K377, move closer to the FAD anion radical, while negatively charged residues, such as D385, exhibit the opposite behavior. The polar residue N391 frequently forms hydrogen bonds with the flavin in the RP_C_ configuration, further contributing to local electrostatic tuning.

The electrostatic potential difference between FAD and Trp_C_ or Trp_D_ is defined as

7a $$\Delta V_{\left(F-C\right)}=V_{\left(r_{F}\right)}-V_{\left(r_{C}\right)}$$

7b $$\Delta V_{\left(F-D\right)}=V_{\left(r_{F}\right)}-V_{\left(r_{D}\right)}$$

These electrostatic potential differences reflect the stabilization of the corresponding radical pair, where a larger positive value of Δ*V*_(F–C)_ (or Δ*V*_(F–D)_) indicates a more favorable environment for electron transfer from the Trp to the FAD. Prior to photoexcitation, as in the DS trajectory, Δ*V*_(F–C)_ and Δ*V*_(F–D)_ are comparable, each approximately 1.1 V (Table 1). However, both Δ*V*_(F–C)_ and Δ*V*_(F–D)_ increase substantially to over 5.5 V for the well-separated RP_C_ and RP_D_ radical pairs, with significant contributions from both the protein environment and the solvent. Solvent plays a more significant role in stabilizing RP_D_ than it does in stabilizing RP_C_, presumably due to the proximity of Trp_D_ to the protein surface. We denote the shifts in Δ*V*_(F–C)_ and Δ*V*_(F–D)_ due to charge transfer as

8a $$\Delta \Delta V_{\left(F-C\right)}=\Delta V_{\left(F-C\right)}^{{RP}_{C}}-\Delta V_{\left(F-C\right)}^{DS}$$

8b $$\Delta \Delta V_{\left(F-D\right)}=\Delta V_{\left(F-D\right)}^{{RP}_{D}}-\Delta V_{\left(F-D\right)}^{DS}$$

As shown in Figure 6B, eight key residues, namely, I316, C317, K320, R356, T364, K377, D385, and N391, account for 77% of the protein reorganization contributing to ΔΔ*V*_(F–C)_. The spatial arrangement of these residues is depicted in Figure 7B. Near the isoalloxazine ring of the flavin, the positively charged guanidinium group of R356 tends to move closer to the FAD, while the negatively charged carboxylate group of D385 tends to move farther away following electron transfer from Trp_C_ to FAD. The largest contribution (0.35 V) arises from N391, which is located in the vicinity of the FAD. The amide group –CONH_2_ of N391 tends to reorient toward the flavin (see Figure 7B), forming hydrogen bonds with the N5 and/or O4 atoms of the flavin moiety (see Table 2), thereby stabilizing the FAD^•–^ radical through favorable electrostatic interactions and partial delocalization of the electron density.

**Table 2:** Hydrogen-Bonding Interactions Involving N391 and the FAD Analyzed from the RP_C_ and RP_D_ Trajectories^a^

	N391(ND2)-FAD(O4) (%)	N391(ND2)-FAD(N5) (%)
RP_C_	21.7	16.8
RP_D_	3.8	1.1

a A hydrogen bond is defined as the hydrogen-bonding angle greater than 135° and the donor–acceptor distance less than 3.2 Å. The percentages refer to the fraction of conformations exhibiting the hydrogen-bonding interaction throughout the trajectory.

Figure 6C, which depicts residue-specific contributions to ΔΔ*V*_(F–D)_, is less structured, suggesting that the stabilization of RP_D_ involves coordinated movement of more residues compared to that of RP_C_. It is noteworthy that N391 does not play a particularly pronounced role in modulating the electrostatic potential for RP_D_, in contrast to its prominent contribution in stabilizing RP_C_. This difference is also reflected in the significantly lower percentage of hydrogen bonding between flavin and N391 in the RP_D_ configuration (Table 2). The terminal residue L495 tunes the electrostatic potential through the movement of the negatively charged carboxylate group –COO^–^, as is evident in the stabilization of RP_D_ (Figure 6C) but not of RP_C_ (Figure 6B). Note that although not depicted in Figure 6B,C, the movement of the negatively charged pyrophosphate moiety of FAD contributes about −0.28 V to either ΔΔ*V*_(F–C)_ or ΔΔ*V*_(F–D)_, which plays a role in destabilizing RP_C_ and RP_D_.

This electrostatic analysis provides guidance for further mutation studies to test the idea of the joint contributions of two well-separated flavin−tryptophan radical pairs to magnetoreception in *Er*Cry4a. For example, mutating positively charged K320 to a neutral residue could disrupt the balance of the electron transfer between Trp_C_ and Trp_D_, thereby altering the equilibrium between RP_C_ and RP_D_. Such a shift in the relative populations of RP_C_ and RP_D_ may lead to appreciable change in the magnetic field effects,^26,47^ which could eventually be assessed through *in vitro* photochemical experiments. Moreover, substituting N391 with an amino acid unable to hydrogen bond effectively is expected to impair the stabilization of RP_C_ more than the stabilization of RP_D_. This destabilization of RP_C_ could increase the energy gap between CT_C_ and charge-neutral states, thereby shifting this radical recombination away from the activationless regime. Such an effect may slow radical recombination of RP_C_, potentially challenging its role in magnetic sensing.

## Concluding Remarks

This theoretical study provides insights into the conformational changes and corresponding electrostatic effects associated with the well-separated radical pairs involving Trp_C_ or Trp_D_ in the *Er*Cry4a protein. Our QM/MM TDA-TDDFT calculations indicate that the flavin−tryptophan radical pairs are stabilized by reorganization of both the protein and solvent to the extent that they can attain energies comparable to those of the charge-neutral state. The combined protein and solvent reorganization can preferentially stabilize the hole on Trp_C_ or Trp_D_, suggesting that conformational fluctuations can facilitate equilibration between these two radical-pair states.

Our QM/MM CDFT-CI calculations were used to compute the free energy profiles and associated electronic couplings for electron transfer between Trp_C_ and Trp_D_, as well as the recombination of the RP_C_ and RP_D_ radical pairs. These calculations suggest that electron transfer between Trp_C_ and Trp_D_ is efficient, occurring on a time scale faster than the coherent singlet–triplet interconversion in both radical pairs. Furthermore, although the recombination of both radical pairs proceeds with a negligible reaction barrier, the electronic couplings for the long-range back electron transfer reactions are very small, leading to slow recombination rate constants.

The approximate time scales estimated in this work are consistent with a geomagnetic field effect arising from radical-pair spin dynamics, which requires a radical pair lifetime on the order of 1 μs (i.e., the Larmor period of the Earth’s magnetic field). However, for an optimal magnetic sensor, the lifetime must also be comparable to or shorter than the spin relaxation time, which was estimated to be several microseconds in cryptochromes.^51,52,78,79^ Our simulations suggest that RP_C_ is more likely to meet this time scale requirement, indicating that it plays a crucial role in magnetic sensing, whereas magnetic sensing based solely on RP_D_ appears less viable. Instead, RP_D_, with its likely lifetime of hundreds of microseconds, may be better suited to stabilizing the signaling state. Together, these results provide theoretical support for the previously proposed ‘composite’ radical-pair magnetoreception^26,47^ enabled by rapid interconversion between RP_C_ and RP_D_.

To provide further insights, our electrostatic analysis identified key conformational changes that stabilize the radical pairs and facilitate electron transfer between Trp_C_ and Trp_D_. Solvent contributes more significantly to the stabilization of the cation radical localized on Trp_D_ compared to the cation radical localized on Trp_C_, presumably because Trp_D_ is positioned closer to the protein surface than Trp_C_. Among the key residues influencing hole transfer between Trp_C_ and Trp_D_, positively charged K320 plays the most prominent role in modulating the local electrostatic potential. Unique to the stabilization of RP_C_, N391 makes a critical contribution through hydrogen bonding to the flavin. Our prediction is that mutations at these sites, as well as at other sites identified in this analysis, could alter the chemical dynamics of the well-separated radical pairs in *Er*Cry4a and lead to changes in magnetic field effects.

## Supplementary Material





## Data Availability

Additional data are provided at the following link: 10.57782/DO4QAY.

## Notes

Lowest 20 excited states obtained from TDA-TDDFT for three representative conformations selected from DS, RP_C_, and RP_D_ trajectories; comparison between TDA-TDDFT and ΔSCF excitation energies; details on free energy profile construction; and individual contributions of residues 8–495 to electrostatic potential differences (PDF)
Input files for quantum chemistry calculations, TDDFT excited-state energies, CDFT-CI matrix for diabatic state energies and electronic couplings, electrostatic potentials, and parameters and initial and final coordinates in MD simulations (ZIP)
