# Supplementary material for: Protein and Solvent Reorganization Drives Radical Pair Stability in Avian Cryptochrome 4a
Source: J Am Chem Soc. 2025 Nov 14;147(47):43934–45. doi: 10.1021/jacs.5c15726 (PMC12673606; doi:10.1021/jacs.5c15726)
Supplement: Supplementary file 1 [file ja5c15726_si_001.pdf]

Supporting Information

Protein and Solvent Reorganization Drives Radical Pair Stability in Avian Cryptochrome 4a

Jiate Luo[1], Jonathan Hungerland[2], Ilia A. Solov'yov[*,2,3,4], Joseph E. Subotnik[*,1], and Sharon Hammes-Schiffer[*,1]

[1]Department of Chemistry, Princeton University, Princeton, New Jersey 08544, United States,
[2]Institut für Physik, Carl von Ossietzky-Universität Oldenburg, Carl-von-Ossietzky Str. 9-11, Oldenburg D-26129, Germany,
[3]Research Center for Neurosensory, Science, Carl von Ossietzky Universität Oldenburg, 26111 Oldenburg, Germany,
[4]Center for Nanoscale Dynamics (CENAD), Institute of Physics, Carl von Ossietzky Universität Oldenburg, 26129 Oldenburg, Germany

*E-mail: shs566@princeton.edu; subotnik@princeton.edu; ilia.solovyov@uni-oldenburg.de

Table of Contents

A. TDDFT Excitation Energies S2

B. Comparison of TDDFT with ΔSCF S3

C. Solvent Exposure S4

D. Free Energy Profiles S5

E. Protein Electrostatic Environment S7

References S7

A. TDDFT Excitation Energies

Table S1: Lowest Twenty Excitation Energies (eV) and Oscillator Strengths[a] from TDA-TDDFT/ωB97X-D/6-31+G** Calculations of Active Site of *Er*Cry4a in Gas Phase or Including Electrostatic Environment of Protein and Aqueous Solvent for DS, RP_C and RP_D Configurations

	**Gas Phase**			**Electrostatic Environment**		
**state**	DS	RP_C	RP_D	DS	RP_C	RP_D
S_1	2.89 (0.05)[b]	3.08 (0.36)[b]	2.96 (0.07)[b]	3.01 (0.07)[b]	0.60 (0.00)[e]	0.41 (0.00)[f]
S_2	3.21 (0.29)	3.14 (0.02)	3.10 (0.36)	3.21 (0.20)	1.52 (0.00)	0.95 (0.00)
S_3	3.49 (0.00)	3.36 (0.00)	3.34 (0.00)	3.33 (0.01)	1.88 (0.00)[f]	1.32 (0.00)[e]
S_4	3.78 (0.00)[c]	3.67 (0.00)[c]	3.67 (0.00)[d]	3.60 (0.00)	2.20 (0.00)[d]	1.77 (0.00)
S_5	4.06 (0.17)	3.91 (0.18)	3.95 (0.15)	3.81 (0.00)[c]	2.25 (0.00)[c]	1.92 (0.00)[c]
S_6	4.22 (0.01)	4.12 (0.00)	4.01 (0.04)	3.85 (0.00)[d]	2.61 (0.00)	2.02 (0.00)[d]
S_7	4.26 (0.00)	4.31 (0.01)	4.13 (0.00)[d]	4.02 (0.21)	2.69 (0.00)	2.66 (0.00)
S_8	4.29 (0.00)	4.40 (0.00)[d]	4.36 (0.02)	4.21 (0.00)[e]	2.91 (0.00)	2.66 (0.00)
S_9	4.38 (0.00)[d]	4.42 (0.00)	4.39 (0.00)	4.31 (0.00)[f]	2.98 (0.00)	2.72 (0.00)
S_{10}	4.58 (0.04)	4.53 (0.00)[e]	4.41 (0.00)	4.33 (0.01)	3.06 (0.23)[b]	2.86 (0.00)[g]
S_{11}	4.68 (0.00)[e]	4.61 (0.00)	4.60 (0.00)[e]	4.48 (0.02)	3.20 (0.00)	2.94 (0.00)
S_{12}	4.80 (0.07)	4.70 (0.00)[f]	4.76 (0.00)[f]	4.62 (0.00)	3.33 (0.00)[g]	3.06 (0.17)[b]
S_{13}	4.83 (0.59)	4.72 (0.14)	4.90 (0.19)	4.63 (0.07)	3.41 (0.03)	3.26 (0.00)
S_{14}	4.84 (0.10)	4.82 (0.10)	4.92 (0.06)	4.66 (0.02)	3.52 (0.00)	3.27 (0.10)
S_{15}	4.84 (0.00)[f]	4.86 (0.90)	4.94 (0.15)	4.84 (0.07)	3.61 (0.00)	3.42 (0.00)
S_{16}	4.90 (0.04)	4.91 (0.05)	4.99 (0.03)	4.87 (0.00)	3.75 (0.24)	3.63 (0.00)
S_{17}	4.95 (0.06)	4.92 (0.03)	4.99 (0.75)	4.90 (0.16)	3.96 (0.00)	3.65 (0.00)
S_{18}	5.02 (0.35)	4.98 (0.15)	5.03 (0.00)	4.91 (0.00)	3.97 (0.00)	3.67 (0.30)
S_{19}	5.04 (0.04)	5.00 (0.08)	5.05 (0.10)	4.91 (0.04)	3.98 (0.00)	3.70 (0.00)
S_{20}	5.04 (0.05)	5.02 (0.01)	5.08 (0.03)	4.95 (0.82)	4.08 (0.00)	3.96 (0.00)

[a] Oscillator strengths are given in parentheses.
[b] Lowest energy LE transition.
[c] Lowest energy CT_A transition.
[d] Lowest energy CT_B transition.
[e] Lowest energy CT_C transition.
[f] Lowest energy CT_D transition.
[g] Lowest energy CT_Y transition.

B. Comparison of TDDFT with ΔSCF

In linear response TDDFT, if the excited state in question does not lie within the response space of the reference, as can be the case in a charge-transfer system, the TDDFT excitation energies will not be accurate. Moreover, the accuracy of TDDFT is limited for very small energy gaps,[1,2] especially in cases where an excited state is nearly degenerate with the ground state. By contrast, the ΔSCF approach involves independently optimizing each of the excited and ground states. In this case, the first excited state is no longer required to lie within the linear response regime of the ground state, and this method can often more accurately predict small excitation energies.[3]

We performed restricted DFT to obtain the energies of the closed-shell charge-neutral state, and we performed unrestricted DFT with spin multiplicity of 3 to obtain the energies of the triplet open-shell $\mathrm{CT_C}$ state in the $\mathrm{RP_C}$ trajectory (or $\mathrm{CT_D}$ state in the $\mathrm{RP_D}$ trajectory). We define the difference between the unrestricted and restricted DFT energies to be the ΔSCF excitation energy, which represents the energies of the $\mathrm{CT_C}$ (or $\mathrm{CT_D}$) state relative to the charge-neutral state. In Figure S1, although TDDFT yields relative energies that are approximately 0.3 eV larger than those obtained with ΔSCF, both methods produce the same sign for the relative energies. Importantly, both confirm that in the $\mathrm{RP_{C(D)}}$ trajectory, the $\mathrm{CT_{C(D)}}$ state fluctuates between the ground and the first excited state, indicating that the $\mathrm{CT_{C(D)}}$ state is comparable in energy to the charge-neutral state when well-separated radical pairs involving the FAD and $\mathrm{Trp_{C(D)}}$ radicals are stabilized.

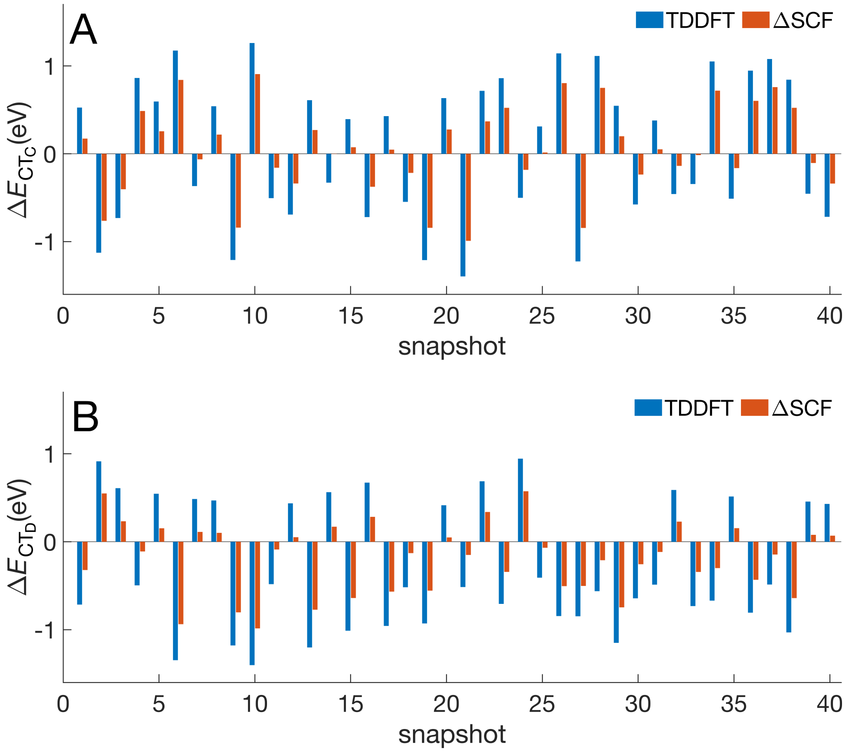

Figure S1: Comparison of the energies of the $\mathrm{CT_C}$ and $\mathrm{CT_D}$ states relative to the charge-neutral state as obtained by TDDFT or ΔSCF for 40 conformations from each of the (A) $\mathrm{RP_C}$ and (B) $\mathrm{RP_D}$ trajectories.

C. Solvent Exposure

The solvent plays a significant role in stabilizing the RP_C and RP_D configurations, as shown in Figure 3 of the main text. Here, we examine the solvent exposure of the flavin and the four tryptophans. As shown in Figure S2, Trp_A and Trp_B exhibit the lowest solvent exposure, the flavin and Trp_C exhibit similar solvent exposure, and Trp_D exhibits slightly higher solvent exposure.

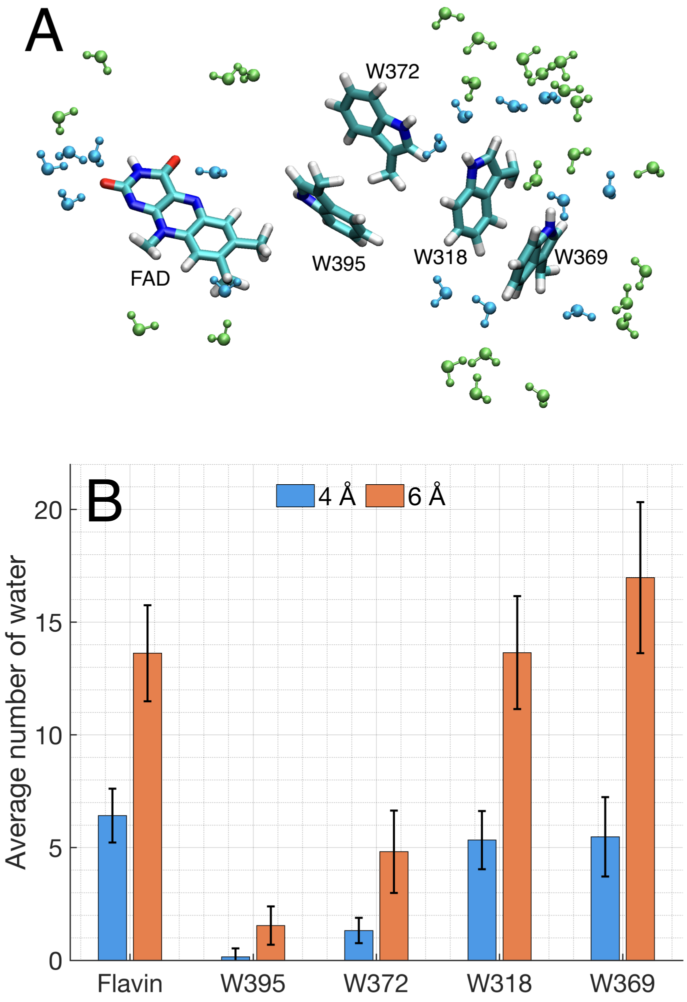

Figure S2: (A) Depiction of water surrounding the flavin and the tryptophan tetrad (W395: Trp_A; W372: Trp_B; W318: Trp_C; W369: Trp_D) for a conformation obtained from the RP_D trajectory. Y319, located at the protein surface and exposed to significantly more water, is omitted for clarity. The water molecules colored blue are within 4 Å of each molecular fragment, while the combination of water molecules colored blue or green represents those within 6 Å of each fragment. (B) The bars show the number of water molecules within the indicated distance from at least one atom of the flavin and tryptophan residues, averaged over the entire RP_D trajectory, with error bars representing the standard deviation.

D. Free Energy Profiles

To study the electron transfer reactions in the well-separated radical pairs, we examined the associated free energy profiles. We extracted 800 conformations at 0.25 ns intervals from each of the MD trajectories equilibrated and propagated for the DS, RP_C, and RP_D configurations. Then, we performed QM/MM CDFT-CI calculations on these conformations to obtain the energies of the diabatic charge-neutral, CT_C, and CT_D states. Gaussian fits were used to obtain the probability density functions of the energy gaps between the relevant diabatic states. We constructed the free energy surfaces as follows:

I. Consider two MD trajectories, each equilibrated and propagated in the reactant and product configurations, respectively. We extracted multiple conformations from each of the reactant and product trajectories. We performed QM/MM CDFT-CI calculations on these conformations to obtain the energies of the diabatic reactant and product states. For conformations from the reactant trajectory, the energy of the reactant state is denoted as $E_{\mathrm{R(R)}}$, and the energy of the product state is denoted as $E_{\mathrm{R(P)}}$, yielding the energy gap, $\Delta E_{\mathrm{R}} = E_{\mathrm{R(R)}} - E_{\mathrm{R(P)}}$. For conformations from the product trajectory, the energy of the reactant state is denoted as $E_{\mathrm{P(R)}}$, and the energy of the product state is denoted as $E_{\mathrm{P(P)}}$, yielding the energy gap, $\Delta E_{\mathrm{P}} = E_{\mathrm{P(R)}} - E_{\mathrm{P(P)}}$. Gaussian fits to the distributions of ΔE_{R} and ΔE_{P} provide the respective averages, $\langle \Delta E_{\mathrm{R}} \rangle$ and $\langle \Delta E_{\mathrm{P}} \rangle$, and variances, σ_{R}^2 and σ_{P}^2. We denote the probability density functions as $P(\Delta E_{\mathrm{R}})$ and $P(\Delta E_{\mathrm{P}})$.

II. Using

$$G(\Delta E) = -k_{\mathrm{B}}T \ln\big(P(\Delta E)\big) + G^{\circ}, \tag{S1}$$

the parabolic free energy surfaces for the reactant and product states are expressed as

$$G_{\mathrm{R}}(\Delta E) = \frac{k_{\mathrm{B}}T}{2\sigma^2}(\Delta E - \langle \Delta E_{\mathrm{R}} \rangle)^2 + G_{\mathrm{R}}^{\circ}, \tag{S2a}$$

$$G_{\mathrm{P}}(\Delta E) = \frac{k_{\mathrm{B}}T}{2\sigma^2}(\Delta E - \langle \Delta E_{\mathrm{P}} \rangle)^2. \tag{S2b}$$

where we set $G_{\mathrm{P}}^{\circ} = 0$. Assuming that the variances of the energy gap distributions for the reactant and product states are approximately equal, the average variance, $\sigma^2 = (\sigma_{\mathrm{R}}^2 + \sigma_{\mathrm{P}}^2)/2$, can be used in these expressions.

III. Given that the parabolas in Eq. (S2) should cross at $\Delta E = 0$, the reaction free energy $\Delta G_{\mathrm{r}}^{\circ}$ (i.e., the change in free energy for a reaction from the reactant state to the product state) and the reaction reorganization energy λ_{r} (reported in the main text) are

$$-\Delta G_{\mathrm{r}}^{\circ} = G_{\mathrm{R}}^{\circ} = \frac{k_{\mathrm{B}}T}{2\sigma^2}(\langle \Delta E_{\mathrm{P}} \rangle^2 - \langle \Delta E_{R} \rangle^2), \tag{S3a}$$

$$\lambda_{\mathrm{r}} = G_{\mathrm{P}}(\langle \Delta E_{\mathrm{R}} \rangle) = \frac{k_{\mathrm{B}}T}{2\sigma^2}(\langle \Delta E_{\mathrm{P}} \rangle - \langle \Delta E_{\mathrm{R}} \rangle)^2. \tag{S3b}$$

The reaction barrier is:[4]

$$\Delta G_{\mathrm{r}}^{\ddagger} = \frac{(\Delta G_{\mathrm{r}}^{\circ} + \lambda_{\mathrm{r}})^2}{4\lambda_{\mathrm{r}}}. \tag{S4}$$

IV. Importantly, within the standard Marcus picture, where we assume the system reaches full equilibrium, the reaction free energy and reorganization energy can be obtained directly from the averages of the energy gaps:

$$-\Delta\tilde{G}^\circ = \frac{1}{2}(\langle\Delta E_\mathrm{P}\rangle + \langle\Delta E_\mathrm{R}\rangle), \tag{S5a}$$

$$\tilde{\lambda} = \frac{1}{2}(\langle\Delta E_\mathrm{P}\rangle - \langle\Delta E_\mathrm{R}\rangle), \tag{S5b}$$

where $\tilde{\lambda}$ is the so-called Stokes-shift reorganization energy.[4] In the limit of ergodic sampling, where all essential conformations have been captured on the timescale of an MD simulation, we would expect

$$\tilde{\lambda} = \lambda_\mathrm{r}. \tag{S6}$$

Refs.[4,5] have argued that the significant nonergodicity of the conformational sampling within proteins, stemming from some unreachable parts of conformational space imposed by the protein structure, leads to $\tilde{\lambda} > \lambda_\mathrm{r}$. For a detailed discussion of ergodicity breaking, see Refs.[4,5]

Electron transfer between $\mathbf{Trp_C}$ and $\mathbf{Trp_D}$: The energy gaps are defined between the $\mathrm{CT_D}$ and $\mathrm{CT_C}$ states as $\Delta E = E_{\mathrm{CT_D}} - E_{\mathrm{CT_C}}$. Gaussian fits provide the average and variance of the energy gaps for conformations sampled from each of the $\mathrm{RP_C}$ ($\langle\Delta E_{\mathrm{RP_C}}\rangle$, and $\sigma^2_{\mathrm{RP_C}}$) and $\mathrm{RP_D}$ ($\langle\Delta E_{\mathrm{RP_D}}\rangle$, and $\sigma^2_{\mathrm{RP_D}}$) trajectories. The corresponding data are shown in Table S2.

Radical recombination of $\mathbf{RP_C}$: The energy gaps are defined between the $\mathrm{CT_C}$ and charge-neutral states as $\Delta E = E_{\mathrm{CT_C}} - E_\mathrm{neutral}$. Gaussian fits provide the average and variance of the energy gaps for conformations sampled from each of the $\mathrm{RP_C}$ ($\langle\Delta E_{\mathrm{RP_C}}\rangle$, and $\sigma^2_{\mathrm{RP_C}}$) and DS ($\langle\Delta E_\mathrm{DS}\rangle$, and σ^2_DS) trajectories. The corresponding data are shown in Table S3.

Radical recombination of $\mathbf{RP_D}$: The energy gaps are defined between the $\mathrm{CT_D}$ and charge-neutral states as $\Delta E = E_{\mathrm{CT_D}} - E_\mathrm{neutral}$. Gaussian fits provide the average and variance of the energy gaps for conformations sampled from each of the $\mathrm{RP_D}$ ($\langle\Delta E_{\mathrm{RP_D}}\rangle$, and $\sigma^2_{\mathrm{RP_D}}$) and DS ($\langle\Delta E_\mathrm{DS}\rangle$, and σ^2_DS) trajectories. The corresponding data are shown in Table S4.

Table S2: Data from free energy profiles for the electron transfer between $\mathrm{Trp_C}$ and $\mathrm{Trp_D}$

$\langle\Delta E_{\mathrm{RP_C}}\rangle$	$\langle\Delta E_{\mathrm{RP_D}}\rangle$	$\sigma_{\mathrm{RP_C}}$	$\sigma_{\mathrm{RP_D}}$	σ	λ_r	$\Delta G^\circ_\mathrm{r}$	$\Delta G^\ddagger_\mathrm{r}$	$\tilde{\lambda}$
22.88	−22.08	6.95	6.63	6.79	13.50	−0.24	3.25	22.48

Table S3: Data from free energy profiles for radical recombination of $\mathrm{RP_C}$

$\langle\Delta E_{\mathrm{RP_C}}\rangle$	$\langle\Delta E_\mathrm{DS}\rangle$	$\sigma_{\mathrm{RP_C}}$	σ_DS	σ	λ_r	$\Delta G^\circ_\mathrm{r}$	$\Delta G^\ddagger_\mathrm{r}$	$\tilde{\lambda}$
2.62	95.55	11.49	9.69	10.63	23.56	−24.88	0.019	46.47

Table S4: Data from free energy profiles for radical recombination of $\mathrm{RP_D}$

$\langle\Delta E_{\mathrm{RP_D}}\rangle$	$\langle\Delta E_\mathrm{DS}\rangle$	$\sigma_{\mathrm{RP_D}}$	σ_DS	σ	λ_r	$\Delta G^\circ_\mathrm{r}$	$\Delta G^\ddagger_\mathrm{r}$	$\tilde{\lambda}$
−2.47	96.69	11.03	10.91	10.97	25.16	−23.90	0.016	49.58

E. Protein Electrostatic Environment

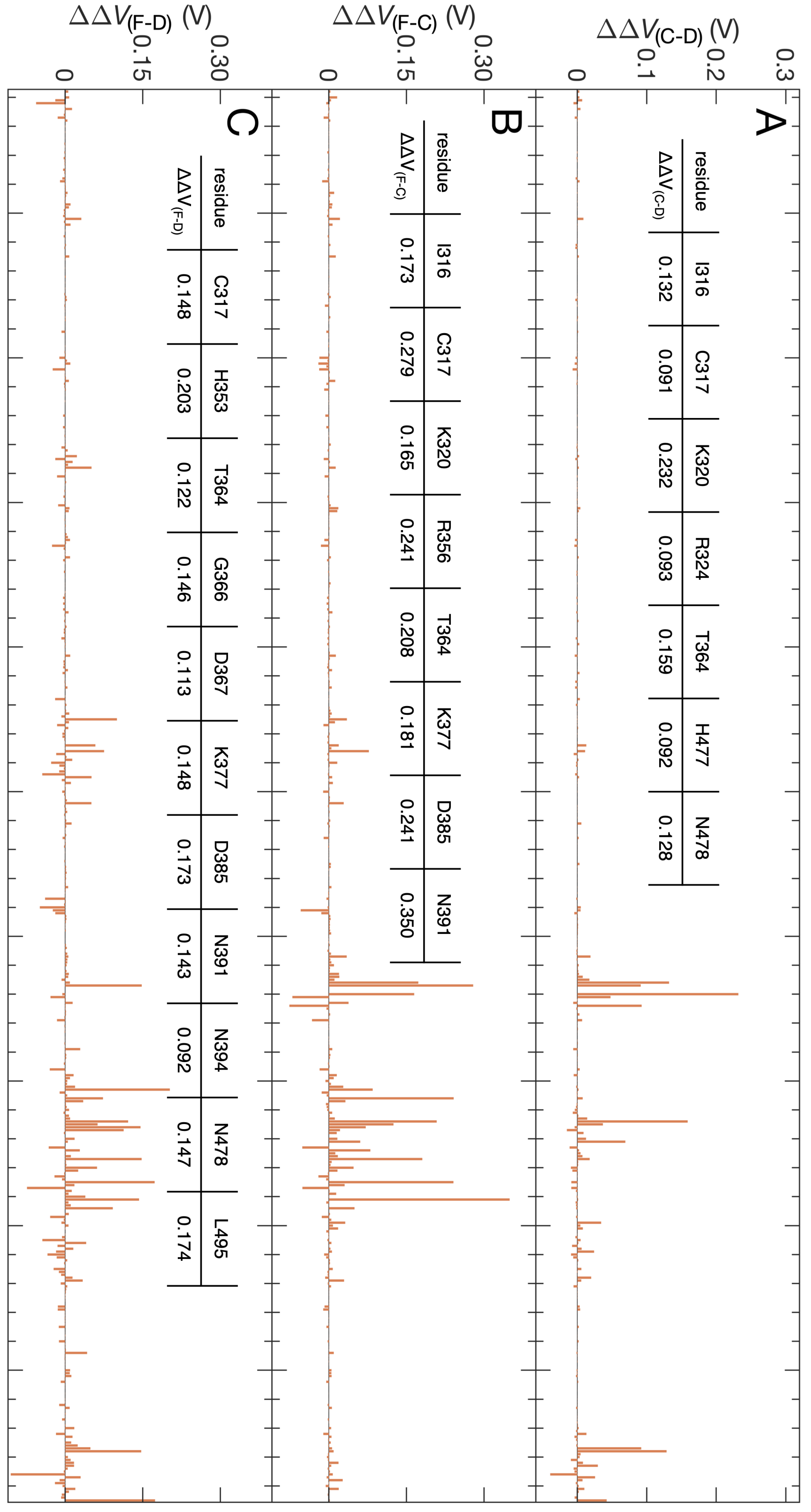

(A)

residue	I316	C317	K320	R324	T364	H477	N478
$\Delta\Delta V_{(C\text{-}D)}$	0.132	0.091	0.232	0.093	0.159	0.092	0.128

(B)

residue	I316	C317	K320	R356	T364	K377	D385	N391
$\Delta\Delta V_{(F\text{-}C)}$	0.173	0.279	0.165	0.241	0.208	0.181	0.241	0.350

(C)

residue	C317	H353	T364	G366	D367	K377	D385	N391	N394	N478	L495
$\Delta\Delta V_{(F\text{-}D)}$	0.148	0.203	0.122	0.146	0.113	0.148	0.173	0.143	0.092	0.147	0.174

Figure S3. Contribution from residues 8 to 495 to the change in the electrostatic potential difference for (A) the hole transferring from Trp_C to Trp_D; (B) the stabilization of RP_C relative to the DS; and (C) the stabilization of RP_D relative to the DS. The tables in the figure show the contribution values of the most influential residues.

References

[1] Levine, B. G.; Ko, C.; Quenneville, J.; MartÍnez, T. J. Conical Intersections and Double Excitations in Time-Dependent Density Functional Theory. *Mol. Phys.* **2006**, *104*, 1039–1051.

[2] Dreuw, A.; Head-Gordon, M. Single-Reference ab initio Methods for the Calculation of Excited States of Large Molecules. *Chem. Rev.* **2005**, *105*, 4009–4037.

[3] Bourne Worster, S.; Feighan, O.; Manby, F. R. Reliable Transition Properties from Excited-State Mean-Field Calculations. *J. Chem. Phys.* **2021**, *154*, 124106.

[4] Matyushov, D. V. Reorganization Energy of Electron Transfer. *Phys. Chem. Chem. Phys.* **2023**, *25*, 7589–7610.

[5] Matyushov, D. V. Protein Electron Transfer: is Biology (Thermo) Dynamic? *J. Phys. Condens. Matter* **2015**, *27*, 473001.
